# Using positive deviance to enhance HIV care retention in South Africa: development of a compassion-focused program to improve the staff and patient experience

**DOI:** 10.1186/s44263-025-00123-3

**Published:** 2025-02-06

**Authors:** Allison J. Ober, Donald H. Skinner, Laura M. Bogart, Leletu Busakwe, Wadene Davids, Hassan Mahomed, Debbie Ling, Virginia Zweigenthal

**Affiliations:** 1 RAND, Santa Monica, CA USA; 2 Department of Global Health, Stellenbosch University, Cape Town, South Africa; 3 Charles R. Drew University of Medicine and Science, Los Angeles, CA USA; 4 Western Cape Department of Health and Wellness, Cape Town, South Africa; 5 Department of Social Work, Monash University, Melbourne, Australia; 6 University of Cape Town, Cape Town, South Africa

**Keywords:** ART/ARV retention, HIV care, Positive deviance, South Africa, Compassionate care, Patient-centered care

## Abstract

**Background:**

HIV burden remains high in South Africa despite intensive efforts to curtail the epidemic. Public primary care facilities, where most people with HIV (PWH) in South Africa receive treatment, face myriad challenges retaining patients on antiretroviral therapy (ART). Nevertheless, some facilities manage to consistently retain PWH in care. We used a participatory positive deviance (PD) approach to discover characteristics of primary care facilities with above-average 12-month retention rates to develop an intervention.

**Methods:**

We conducted 11 in-depth leadership interviews, 9 staff focus groups with 29 participants, 11 patient focus groups with 45 participants, 23 patient shadowing visits, and multiple facility observations in each of 3 high- and 3 low-retention public primary care facilities in Cape Town, South Africa. Using PD, an asset-based approach to behavior change that consists of discovering how high-performing outliers succeed despite sizable barriers, and then using those data to develop interventions for low performers, we analyzed data to discover dominant characteristics of higher-retention facilities that might be contributing to higher retention rates.

**Results:**

Dominant themes found in higher-retention facilities were compassionate, respectful, and patient-centered care; higher staff morale, passion for the work, and team cohesion; efficient workflow procedures; and a welcoming physical environment. From these themes, we developed the multidimensional Connect intervention, consisting of strategies within three domains: (1) engage, encourage, and support staff (e.g., a monthly staff support huddle, a compassion training); (2) expedite and augment workflow procedures (e.g., adjust folder system to lower wait times); and (3) create a welcoming physical environment (e.g., fresh paint and plants in the waiting area).

**Conclusions:**

A PD approach enabled us to identify factors that could be contributing to higher ART retention rates within low-resource public sector primary care facilities in Cape Town, South Africa, and to develop a multidimensional intervention. If effective after a future trial, the intervention could be a feasible, affordable complement to existing programs aimed at improving care for PWH.

**Supplementary Information:**

The online version contains supplementary material available at 10.1186/s44263-025-00123-3.

## Background

HIV burden remains high in South Africa despite intensive efforts to curtail the epidemic. The country has the largest HIV epidemic globally, with 7.6 million children and adults living with HIV, and a national prevalence among those 15 to 49 of 17.8% [1]. South Africa has made some progress toward the UNAIDS 95–95-95 goals of 95% of all people with HIV (PWH) diagnosed, 95% of those with a positive HIV diagnosis on antiretroviral therapy (ART), and 95% of those on ART with undetectable HIV RNA (i.e., viral load suppression (VLS)) by 2025. In 2022, 94% of those with HIV had been diagnosed, 75% of PWH were receiving ART, and 69% were virally supressed [1]. Retention in care has remained an ongoing challenge [2, 3], with fewer than 60% of PWH starting ART remaining in care at the same facility with continuous care for more than 28 days and 6 months after they initiated ART, as of 2018 [4]. While some patients (about 11%) who disengaged from their initiating facility transferred to different facilities and some reentered care cyclically (14%), many did not return at all (58%) [4]. The initial 6-month engagement period is vital because it is associated with remaining in care for 12 months and beyond after ART initiation [4].

Public sector primary care facilities, where most South African PWH receive ART for HIV treatment at no cost, face myriad challenges to retaining patients long enough to achieve viral suppression. Ongoing, pervasive barriers to service delivery are at the patient, clinic, and health-system levels. Patients experience individual and social challenges that interfere with their ART adherence, such as substance use [5, 6]; stigma, which can limit treatment due to patients wanting to hide their diagnosis [7]; and, relatedly, poor social support [8, 9]. At the clinic level, often driven by low resources within the health system, there are staffing shortages [10], long queues [11], inconvenient clinic hours [11], medication stock-outs [12, 13], poorly trained adherence counsellors [14–16], and poor service quality and communication among health care providers [16, 17], all of which cause disruptions in ART adherence and retention in care for PWH. Additional clinic-level barriers experienced by PWH include stigma and discrimination within the facility [11, 18–20], unfamiliar facility environments [18], lack of confidentiality [20], and visits scheduled on different days for different conditions [18]. In addition to these barriers, PWH in South Africa also face structural challenges that impede ART adherence and retention in care, such as food insecurity [21, 22], distance to facilities, and lack of finances for travel [12, 22]. Although evidence-based programs and practices such as adherence clubs [23] can increase PWH adherence and retention, implementing and sustaining programs and practices in crowded, overburdened health care settings remain a challenge.

Despite pervasive barriers, some public sector primary care facilities manage to consistently retain PWH in care. Many clinics with high retention rates are comparable to lower-retention clinics, with similar resources, patient demographic characteristics, and number of patients, and they face the same multi-level resource and capacity limitations. Although there is a large literature on barriers and facilitators to ART adherence and retention in care, there is little information about what clinics that perform well are doing to retain patients in care after they initiate ART.

We used a positive deviance (PD) methodological approach to discover characteristics of or strategies used by higher-retention facilities in the Western Cape to develop and test an intervention aimed at improving ART retention. PD is an asset-based approach to individual and organizational behavior change that consists of discovering ways in which high-performing outliers manage to succeed despite sizable barriers and then sharing successful strategies with lower performers to improve outcomes [24, 25]. PD is based on the observation that typically some individuals or groups find better solutions to problems than others who have access to the same resources yet face similar challenges [25]. “Deviance’ in the context of PD refers to outcomes and/or behaviors that deviate from the norm [26]. The PD approach involves (1) developing a case definition to operationalize PD for the setting, (2) identifying those who have achieved good outcomes despite high risk, (3) interviewing and observing these individuals or organizations to discover uncommon strategies or behaviors that could explain the good outcome, (4) analyzing findings to confirm that strategies or behaviors are indeed uncommon and determine which could be realistically implemented by those who could benefit from them, and (5) designing and implementing behavior change tools and activities to encourage adoption of the new strategies or behaviors [27].

Within the past decade, the PD approach has emerged as a strategy for improving health services at the organizational level [28–41], such as by identifying high-performing diabetes care facilities to improve care across a health care system [28], reducing hospital emergency room crowding by identifying practices in high-performing department [42], and identifying strategies to improve access to primary care [43]. In this article, we describe intervention development methods and findings from the qualitative work conducted to develop the PD intervention. We partnered with the Western Cape Department of Health and Wellness (WCDHW) as well as health system, primary care facility, and patient stakeholders to inform and participate in intervention development and implementation throughout the study. Effects of a pilot test of the intervention on retention rates, along with implementation feasibility, will be described in a subsequent article.

## Methods

### Study overview

We conducted semi-structured interviews with facility leaders and focus groups with providers and PWH and patient shadowing to discover strategies used by primary health care facilities managing to retain PWH in care despite pervasive challenges. We then analysed the data to develop a PD intervention that consists of a manual with novel PD strategies and methods for implementing PD strategies to be sustainable.

### Study setting

Data collection took place between June 2021 and September 2023 within provincially administered health facilities governed by the WCDHW. At the start of data collection, the Western Cape province public sector system was comprised of 447 primary care service points across 6 districts. For this study, we selected from provincially run Cape Town District primary care facilities that provide HIV services in an outpatient setting. We excluded hospitals and specialized services, such as correctional services. The focus was on “community health centers” (CHC, which provide 24-h services) and “community day centers” (CDC, which provide services for 8 h daily) within the City of Cape Town Metropolitan Health District that are managed by WCDHW (*N* = 40 at the start of our study). The reason for restricting our study to primary health care facilities within WCDHW for this study was to select facilities with similar resources facing similar challenges. For example, within Cape Town, there are fewer transportation barriers for patients to attend clinic visits, with a facility located within at most five kilometers of most residential areas and formal and informal settlements (i.e., areas where displaced populations settle outside of urban areas or in rural areas). However, in areas outside of Cape Town, transportation and clinic availability are larger barriers. We also omitted tertiary hospitals and health facilities run by other management authorities, such as correctional services or the municipality of the City of Cape Town, to limit contextual variation that could influence findings.

The facilities provide free services and medication for patients using defined treatment approaches and algorithms. Typically, public health clinics have large numbers of patients, especially in relation to staff numbers. For example, the South African Nursing Council reported that there is 1 nurse for every 236 people in public health clinics in the Western Cape; others report a lack of experienced health professionals in the public health system [44–46]. While some appointments are set in advance, services are typically provided on a first-come, first-served basis. Patients generally arrive early morning before the clinic formally opens to queue and then can sometimes wait up to 6 or 7 h. Some services are provided by lay staff (i.e., community health workers, HIV counsellors), through a range of contracted nongovernmental organizations (NGOs). Lay staff are particularly important for HIV care, as they provide a substantial proportion of the counseling and patient tracking (if patients miss appointments) on behalf of the clinic.

We selected six facilities to participate in PD “discovery”: three higher-retention and three lower-retention facilities, based on those above and below average (59%) for 12-month retention in care in 2018 (see Additional File 1: Table S1). We elected to examine 12-month retention (i.e., the proportion of patients still in care at the same facility 12 months after ART initiation, excluding transfers) for facility selection to ensure ongoing high performance beyond the initial engagement period. We matched facilities based on size (small, medium, large) and other characteristics such as proximity to transportation and patient demographic characteristics (i.e., whether patients are predominantly Black, Coloured, or both). We selected higher- and lower-retention facilities based on data on all patients in all provincial facilities who initiated ART in 2018 who were retained on ART 12 months later; we validated this against 2019 data.

### Stakeholder advisory board

The study was participatory, informed throughout the process by feedback from a Stakeholder Advisory Board (SAB) consisting of study investigators, administrators from within WCDHW, facility managers, nurses, and adherence counsellors and community health workers, as well as PWH from facilities not involved in the study. Most SAB members met three times (twice virtually and once in person) during the intervention development period and provided substantial additional input on the development of the intervention through ad hoc meetings and materials review.

### Procedures

We used qualitative methods to discover unique or uncommon strategies of higher-retention facilities compared with the lower-retention, case–control facilities. We follow recommendations in the 32-item COREQ (COnsolidated criteria for REporting Qualitative research) checklist to report our procedures (see Additional File 2) [47]. All investigators conducting research were trained in qualitative methods and on specific study measures. Research investigators did not have prior relationships with participants. Consent forms described research activities and organizations involved; information about research staff was not discussed.

### Primary care facility leader semi-structured interviews and provider focus groups

#### Qualitative guides

We developed provider interview and focus-group guides consisting of broad open-ended grand tour questions asking about how the facility addresses common barriers to retaining PWH in care, as well as additional questions about how they address barriers in the specific workflow process of the clinic, about possible strategies to improve retention, and about how the clinic manages to maintain a positive workforce climate, teamwork, and empathy (see Additional Files 3 and 4). Similar guides were used for the leadership interview and provider focus groups, with the leadership guide also asking questions around administrative strategies, policies, and standard operating procedures that could be affecting ART retention. We incorporated specific PD probes, informed by the PD field guide [27] to uncover intentional strategies for addressing overall barriers as well as activities, practices, or attitudes that may not be intentionally aimed at improving retention but could inadvertently be supporting it. We provide examples of questions and probes in Additional File 5: Table S2.

### Procedures

We invited each facility leader to participate in a 1-h, in-person interview. For most facilities, an additional interview was done with the leader of the HIV and AIDS, sexually transmitted infections, and tuberculosis (HAST) service and/or the medical doctor involved. We also conducted two provider focus groups at each facility: one for professional staff (nurses, physicians) and the other for lay staff (community health workers, HIV counsellors). Providers were invited through the facility manager and the leaders of the HAST service at each facility. Focus groups were held during times the facility deemed to be least disruptive to service provision. Participants were provided with snacks during the group discussion. We elected to hold individual interviews with facility managers because their presence in focus groups could influence responses of other providers and because we wanted to collect information on strategies at the policy and administrative level that may not be relevant to clinic staff. We conducted separate focus groups for professional and lay staff because responsibilities, training, and power dynamics tend to differ between the groups. Interviews were conducted by researcher investigators D. S. (PhD) (man), Z. P. (PhD) (woman), and research assistant L. B. (BA) (woman).

### Patient focus groups

#### Qualitative guide

We developed a patient focus-group guide that followed a similar format to the provider guide, but we tailored the questions to patient experiences at the facility. Like the provider guide, we started with open-ended “grand tour” questions (see Additional File 6).

#### Procedures

The nursing staff at each facility facilitated the recruitment of patients based on the requirements of the study. This allowed for the identification of patients who would be able to respond adequately and avoided fears of identification by the patients. Eligibility criteria included the following: (1) 18 years of age or older and (2) patient of and retained in care at the clinic for 6 months or longer after initiating ART. Patients were compensated R120 (~ US $8.50) for participation. (Incentive amounts reported in this article reflect approximate exchange rates at the time the study was conducted, between 2021 and 2023.) Focus groups lasted approximately 1 h. Focus groups were conducted by researchers D. S. (PhD) (man) and Z. P. (PhD) (woman) and a research assistant L. B. (BA) (woman).

### Patient shadowing

#### Observation form

We adapted a patient shadowing observation form based on prior research (see Additional File 7) [48–50]. The form included places for the shadowing researchers to record the information during the workflow experience. The period the patient spent in the exam room was not captured, as the shadowing research staff member was not allowed in the exam room. The investigators observed and captured the following: time and duration of all events; who entered and left and what they did and said; location; touch points or anyone who came in contact with the patient; care experience pathway (where did the patient travel within the setting, what was the climate like); and the atmosphere in each space. The shadowing researcher’s notes included observations about what seemed to work well and why. Following the visit, the researcher asked the patient about their impressions of each part of the facility visit, asking about what worked well and what might make them most likely to come back to the facility.

#### Procedures

The nurses in each facility’s HAST service assisted in the recruitment of PWH—two men and two women—from each of the six facilities who had been retained on ART for 6 months or longer. Patients signed up using a first name only. Patients were compensated R150.00 (~ US $8.01). No identifying information was shared with the shadowing researcher. Shadowing researchers met patients at the facility on the day of their appointment and shadowed them as they moved through the facility, from waiting area to exam room, and noted characteristics of the facility and patient interactions. Shadowing lasted 4 h, on average. Research investigators L. B. (BA) (woman) and W. V. (BA) (woman) conducted patient shadowing.

### Researcher observation

#### Measures and data collection

To understand facility structure and processes, all study researchers and staff visited facilities on multiple occasions and took photographs and detailed notes describing the physical environment, surrounding neighborhood, and security features. Photographs included infrastructure only and not patients or staff. Staff recorded field notes after each visit. The focus for observation was patient workflow systems, resources available, and the general atmosphere. The team also ascertained which policies and systems had been implemented and to what extent. Research investigators D. S., A. O., L. M. B., L. B., and W. V. conducted observations.

Sample sizes for interviews, focus groups, patient shadowing activities, and observations at each facility are shown in Table 1.

**Table 1:** Qualitative data collection participation

	**Facility 1** **(HR)**	**Facility 2** **(LR)**	**Facility 3** **(HR)**	**Facility 4 (LR)**	**Facility 5** **(HR)**	**Facility 6 (LR)**
Manager interviews	*N* = 2	*N* = 2	*N* = 1	*N* = 2	*N* = 2	*N* = 2
Staff focus groups	*N* = 1 group, 5 participants	*N* = 2 groups, 3 participants	*N* = 1 group, 3 participants	*N* = 1 group, 3 participants	*N* = 2 groups, 8 participants	*N* = 2 groups, 7 participants
Patient focus groups	*N* = 2 groups, 9 participants	*N* = 1 groups, 4 participants	*N* = 2 groups, 7 participants	*N* = 2 groups, 8 participants	*N* = 2 groups, 7 participants	*N* = 2 groups, 10 participants
Patient shadowing	*N* = 4 (2 men, 2 women)	*N* = 4 (2 men, 2 women)	*N* = 4 (2 men, 2 women)	*N* = 4 (2 men, 2 women)	*N* = 4 (2 men, 2 women)	*N* = 3 (1 man, 2 women)
Facility observations	*N* = 10	*N* = 11	*N* = 11	*N* = 12	*N* = 10	*N* = 11

### Composite analysis of findings to discover PD themes

All interviews, focus groups, and patient shadowing notes were recorded and transcribed. We conducted rapid analysis of the qualitative data to identify potentially actionable insights [51]. Studies have shown that themes generated by rapid versus conventional, in-depth analysis to inform implementation are highly similar [52]. For the rapid analysis, three investigators (A. J. O., D. S., L. M. B.) first read all transcripts and notes, viewed facilities in person, and reviewed photographs taken during visits. Each separately noted themes that emerged from the data and wrote independent summaries of impressions and potential PD strategies that emerged from all facilities. Next, all team members met in person in Cape Town over 3 days for intensive discussion of themes and potential strategies. After a list of PD themes had been developed, the team ruled out themes that were prevalent in both higher- and lower-retention facilities, consistent with the PD framework. The initial outcome of the composite analysis, which triangulated data across all forms of data collection, was a list of overarching domains and specific PD strategies within each domain that could be adapted to fit multiple facilities. Although we did not member-check transcripts of themes with all participants, the team presented the initial domains and strategies in a 4-h SAB meeting to ensure the proposed strategies were feasible to implement and sustain (i.e., to fit with current practices and have clear, demonstrable outcomes) [53]. Following the meeting, the manual was developed and then reviewed and approved by SAB members.

After the intervention was developed, we conducted traditional content analysis [54] to validate our rapid analysis findings and inform further inquiry. For this analysis, the research team (A. O., D. S., L. M. B., L. B.) developed a codebook to categorize emergent themes (see Additional File 8). Using Dedoose (qualitative data management software [55]), the team first entered all domains and subdomain themes into the codebook. Research assistants L. B. and W. V. then marked areas of text pertaining to each domain and construct code. L. B. and W. V. practiced with a random sample of 10% of transcript sections, coding independently and reviewing together. If coder disagreement revealed ambiguity in the codebook, the larger team discussed the disagreement and modified the codebook. Training continued until the two coders could consistently identify and mark each theme. Next, both coders worked on three transcripts independently, after which we measured coder consistency for each theme. Once consistency was reached, evidenced by kappas of ≥ 0.70 is considered “good” consistency [56]; each coded half of the remainder of the transcripts independently and discussed and resolved inconsistencies.

## Results

### Participants

Ninety-nine percent of facility leaders interviewed were women, 36% were Black, 45% were Coloured, and 18% were White. (In South Africa, the word “Coloured” is used to describe people of mixed race who are not White, Black. or Asian. [57]) Eighty-six percent of staff who participated in focused groups were women, 31% were Black, 66% were Coloured, and 4% were White. Patient focus-group participants were 55% women; 86% were Black, and 14% were Coloured. Sixty-six percent of patients who participated in the shadowing exercise were woman; 74% were Black, and 16% were Coloured. Of note, where quotes are provided, to protect participant confidentiality, we combine leader interview and staff focus-group labels, and do not report patient gender.

### Composite analysis findings

Our analysis yielded several dominant PD themes (i.e., those that predominantly emerged in the higher-retention facilities). Below we describe each theme and provide illustrative quotes. We note that theme saturation was achieved, in that by completion of our analysis of all data, themes repeated, with no new themes apparent.

#### Positive patient experience: compassionate, respectful, and patient-centered care

Overall, in the three higher-retention facilities, more than at lower-retention facilities, patients reported receiving exceptionally compassionate, respectful, and personalized care from staff or recounted anecdotes indicative of this type of care, including how good they felt when staff members knew their names and asked about their families. At these higher-retention facilities, staff and patients typically mentioned one or two specific staff members who showed exceptional, individualized care, passion for their jobs, and compassion for their patients. Also, at these facilities, patients and providers alike noted processes of care and experiences in which patients are viewed holistically and treated with empathy and compassion.

The fact that you can talk to the sisters, you can ask if there’s something you’re not happy with and they will gladly assist you. So, I think even that – that availability of staff actually makes it more… you feel more at ease, you feel confident. You feel that you will be helped because they would assist you if you ask. So relationships are also very important. (Patient Shadow, Higher-retention facility)

Staff in higher-retention facilities emphasized that they are dedicated and committed to patients’ overall health and well-being and discussed, more so than staff at lower-retention facilities, the importance of a friendly, nonconfrontational orientation toward patients. There were some comments about negative experiences at higher-retention facilities, generally pertaining to long wait times. During patient shadowing activities at higher-retention facilities, the research investigator noted on several occasions that patients were greeted by name by friendly, warm staff, including security staff.

You know, you don’t judge, we give them a hearing and we try to give them a solution. And how can we assist your life a bit better or your life a bit easier. So we encourage them, tell us; speak to us – we’re human and we are there to walk the journey with them. And they’re not alone on this journey, so we try our best…, we try our best. (Staff Focus Group/Leader Interview, Higher-retention facility)

In comparison, in lower-retention facilities, reports of patient-friendly procedures and patient experiences seemed to be mixed, with some positive anecdotes and sentiments but notably fewer comments indicating enthusiastic feelings about staff, staff passion for their jobs, and positive experiences. At these facilities, there were more negative comments about experiences at the facility than there were in higher-retention facilities. Where in higher-retention facilities patients and staff perceived that patients returned to the facility for care because of kindness and compassion, patients and staff at lower-retention facilities spoke more about patients returning because they receive good, acceptable service. Additionally, in lower-retention facilities, some patients recounted stories of being treated with disrespect and lack of confidentiality about their HIV status, and not feeling welcome. Also, some staff at these facilities reportedly showed a negative, disrespectful attitude toward patients.

Because there does seem to be a lot of cases of clinicians shouting at patients and they’ve never met the clinicians from what… I think there might be fear on the part of the patients? (Staff Focus Group/Leader Interview, Lower-retention facility)

Patients at all facilities, whether higher or lower retention, expressed that not being “shouted at” was among the reasons they return to their facilities, regardless of the facility’s proximity to their work or home. Several patients at both higher- and lower-retention facilities said they left their previous facility because they were treated poorly by staff, often being scolded for making mistakes or returning after falling out of care.

#### Positive staff experience: higher staff morale, team cohesion, and support for staff

Staff morale stood out as better at the higher-retention facilities, with higher staff cohesion, than at the lower-retention facilities. The environment across staff—with other staff and management—seemed to be more collaborative, with staff appearing to feel connected to each other. Staff at higher-retention facilities discussed a collaborative environment in which they “work as a team” in a “partnership” and have a “passion” for their work.

Look, … I think the morale is quite high I mean, we work together quite well as a team and I think it’s, because of the consistency of the staff. I mean, our absenteeism is very low. We enjoy what we do, there’s a passion for what we do. We’ve done this work for a long time, and we have relationships with the patients. (Staff Focus Group/Leader Interview, Higher-retention facility)

In contrast, in lower-retention facilities, staff reported substantially more stress and burnout and requested counseling due to their stress and unsafe conditions. There was less cooperation between staff, or cooperation was more difficult, leaving staff feeling isolated in their roles. They also said that a better relationship with management was needed.

And sometimes as nurses, we really do need counseling. We've got a lot of things that we are being exposed to here in the clinic. We experience trauma. You hear a lot of stories from patients. Depressed patients and the challenges that they face. And when they see you, they see someone they can talk to, that they can get help from. So sometimes we also need that time to just offload whatever we are feeling. But I think there's something lacking when we look at those because we really don't get that. So, some of us had to dodge bullets, stones along the road. So, it was traumatic. It was only then that our Operational manager was trying to organize something for us to get some counseling. (Staff Focus Group/Leader Interview, Lower-retention facility)

#### Efficient workflow procedures: wait times, patient tracking, and reintegration

Long wait times generally were an issue across facilities but seemed to be shorter in higher-retention facilities, possibly due to more efficient workflow procedures. For example, in all higher-retention facilities, folders for the ART patients from the general facility are pulled the day before the patient visit so that they are ready for HIV care visits, and there are designated follow-up procedures. Patients who have been retained in care without problems over at least 6 months are given their antiretroviral medications (ARVs) through a club system that expedites service.

In lower-retention facilities, such procedures were not highlighted; when they were, they seemed to be less clearly defined.

Interviewer: So you do use a booking system as well for people coming in? Participant: Ya, sort of…. It comes and goes. (laughs)

Interviewer: So it’s not as rigorous as you’d like but you’re also different because you see them, if there are immediate referrals, you see them.

Participant: Uhmm… I don’t mind, sister [NAME] got to book them and put the stickers on. I think it’s more for her to remember which patients are coming back. (Staff Focus Group/Leader Interview, Lower-retention facility)

Patients seemed to be better tracked in higher-retention facilities, with more systematic identification of patients who miss appointments. One facility had a flexible system, in which patients who miss appointments are never turned away. In the same facility, a “welcome service”—a non-punitive workflow protocol that facilitates patient reentry into the facility that is supposed to be standard of care within all facilities in the WCDHW)—is consistently implemented for patients who have fallen out of care. In contrast, in a lower-retention facility, patients who show up without an appointment or having missed several appointments discussed sometimes being treated in a punitive way, with tracking and follow-up procedures less clearly defined.

#### Welcoming physical environment

Across multiple facility visits, researchers observed differences in physical environments. In the higher-retention facilities, they noted bright open spaces in patient waiting areas throughout the facility. Two higher-retention facilities had outdoor areas with benches, grass, and plants; all three looked well-maintained, with, for example, freshly painted walls in indoor waiting areas, artworks on the walls, clear signage, and visible patient-oriented materials, such as WCDHW patients’ rights posters and other posters with motivational slogans. One higher-retention facility gave patients access to Wi-Fi. During shadowing exercises, patients told research staff that they sometimes came to the facility just to use the Wi-Fi, even if they did not have an appointment that day. In contrast, lower-retention facilities were generally older buildings and were less well maintained especially due to the areas in which they are located, limited upkeep, and as the need for upgrades due to the crime and safety of the surrounding neighborhoods. Outside most of the facilities were also informal vendors who sold small provisions (e.g., chips, sweets, fruits, cold drinks). Lower-retention facilities appeared more crowded. The outside areas did not have gardens, and most were fenced in with access only by one primary entrance which was guarded by security personal. Any remaining open space was used for parking as well as alternative waiting areas. They had fewer attractive features inside with walls either bare or showing old and outdated posters and minimal instructions for navigating the clinic.

### Participatory intervention development

After reviewing the emergent themes, the research team (A. O., D. S., L. M. B., L. B., W. V., H. M., V. Z.) categorized the most common themes from higher-retention facilities into two overarching themes of (a) positive staff and patient experiences and (b) strong connections among staff and patients. These overarching themes were discussed in a SAB meeting, in which SAB members discussed several intervention strategies that could lead to improved staff and patient experiences and connections between staff and patients to improve patient retention. Proposed strategies included the following: (1) Toward staff cohesion, providing communication training for staff, based on evidence-based motivational interviewing [58] tools and techniques, holding a monthly team meeting in which retention data and strategies are discussed and team-building exercises are conducted; (2) toward efficient workflow procedures, pulling folders for next-day appointments, pre-packing ARVs for next-day appointments, implementing E-lockers, and consistently implementing welcome services; and (3) toward a welcoming physical environment, installing plants, murals, and brightly colored paint, providing patients access to food and water, and installing Wi-Fi.

Stakeholders generally supported the proposed strategies but suggested modifying or eliminating several of them. The SAB emphasized that strategies would need to be realistic and match staff needs and the daily realities of providing treatment in a challenging setting, and that they should be feasible and sustainable in a low-resource environment. We modified or eliminated strategies based on SAB input.

### Connect program domains and strategies

Based on the formative qualitative data and input from the SAB, we developed a multidimensional program of intervention strategies to address several identified themes. We called the program “Connect” to reflect the emphasis on staff-to-staff and staff-to-patient connections. We developed Connect around the emergent themes of positive staff and patient experiences at the facility aimed at improving ART retention, achieved by providing staff support and ultimately improving compassionate, patient-centered care. The program consists of a core domain—engage, encourage and support staff—with two core strategies—monthly staff support huddle and a compassion training. At a minimum, to increase retention, facilities are expected to implement these core strategies, and to consider other related strategies, as feasible for each facility. We describe each domain and strategy below.

### Domain 1: Engage, encourage, and support staff

#### Strategy 1: Monthly staff support huddle

A key component of the monthly huddles is called “Connect Rounds,” which we modeled off the Schwartz Rounds® [59, 60], a method of conducting “grand rounds” (formal meetings during which providers discuss cases) that has been shown to improve compassion toward the self and others, reduce stress, and improve teamwork and openness to change among participating staff [61–63]. Key features of Connect Rounds are as follows: (1) A standard monthly meeting with refreshments provided; (2) facilitation by a senior doctor or nurse who can help presenters prepare, lay ground rules, and contain emotion to allow for safe expression of feelings; (3) presenters, selected the month prior and who prepare in advance, who offer personal stories and perspectives on an agreed-upon theme, scenario, or patient case; and (4) an invitation to the team to share and reflect. Topics are typically nonclinical (e.g., psychosocial, ethical, emotional) issues surrounding the patient-caregiver relationship [60].

#### Strategy 2: Compassion training

Strategy 2 is a compassion training for healthcare workers, which we adapted from an evidence-based compassion training developed by Dr. Debbie Ling of Monash University [64] as well as motivational interviewing-informed tools and techniques [65]. The training is designed to be delivered over two sessions and conveys the essential elements thought to be needed to improve compassionate care. The essential elements of this training are intended to be reinforced during monthly huddles. Compassion in this training is operationalized as a sense of concern for the person suffering combined with the motivation to alleviate the suffering [66]. An important part of the training is distinguishing between compassion and empathy [66]. Empathy is sharing feelings with others and can accidentally lead to “empathic distress” for the worker. Compassion by contrast is focusing on alleviating the other’s suffering and protects against *empathic distress* [67]. Empathic distress is defined as stress and burnout caused by feeling too much empathy [68]. Essential elements of the Connect compassion training, based on prior compassion training research [62], are as follows: (1) communicate to participants that compassion with patients can reduce *empathic distress* and improve healthcare worker wellbeing; (2) describe how compassion differs from empathy in that in addition to empathy’s key feature of “feeling with” another person, compassion adds being motivated to help the person; and (3) emphasize a common humanity orientation (e.g., “just like me, this person wishes to be happy and not to suffer”) to teach healthcare workers the practice of recognizing common humanity in order to foster positive emotions toward patients [69]. The training would be conducted by an experienced WCDHW trainer who is familiar with the facility environment.

### Domain 2: Create a welcoming physical environment

#### Strategy 3: Physical improvements

This strategy consists of engaging the facility team staff in determining what, if any, physical improvements can feasibly be made to the facility. Because of severe budget constraints within the WCDHW, the project provided a small amount of money (R6000/US $220) to each facility to make improvements. The Connect manual offers suggestions for procuring additional funds as well as WCDHW and community support, as any changes in facilities must be approved by the department. Additionally, neighborhoods near some facilities may have active community committees that are involved with facility decisions.

### Domain 3: Expedite and augment workflow processes

#### Strategy 4: Pre-pull folders/hold missed appointment folders for immediate tracking

This strategy consists of changing folder procedures so patient folders are pulled the night before scheduled visits and having a clear protocol for transferring information about missed appointments to the community health worker who can track the patient. This approach also expedites the movement of patients through the service reducing waiting times.

#### Strategy 5: Welcome Back Service

This strategy consists of implementing an existing “Welcome Back Service” policy for individuals who have fallen out of care [70]. Welcome Back Services are an example of an existing program or policy meant to be implemented by all facilities that may not be implemented fully or at all. Welcome Back Services typically include standard operating procedures for handling patients who reenter care, enhanced counseling provided by facility staff as well as peer-lead counseling, education materials, and staff training on providing care that helps empower patients who return to care. For our framework, we drew on Welcome Back Service manuals [3] developed by the Department of Health South African and Médecins Sans Frontières (MSF), an international, independent, medical humanitarian organization, who developed and implemented this in public sector facilities in Cape Town.

## Discussion

Employing a participatory PD approach [24, 25], we conducted comprehensive qualitative research to uncover distinguishing characteristics and strategies of primary care public sector health facilities in Cape Town, South Africa, which exhibit above-average HIV care retention rates despite operating under low-resource conditions. Our study revealed a compelling overarching theme: facilities with higher retention rates consistently prioritize a positive experience for both staff and patients, emphasizing compassionate, patient-centered care. Consistent with this theme, we identified and documented strategies—either actively employed by higher-retention facilities or evidence-based practices deemed feasible and sustainable by key stakeholders—which could be adapted to similar contexts. This led to the development of the “Connect” program, which we subsequently evaluated in a pilot study of lower-retention facilities within the same health system (WCDHW) for its feasibility, acceptability, and preliminary impact on retention rates (findings forthcoming).

Consistent with Connect’s compassion-based strategy, a growing body of literature suggests that a focus on providing space for healthcare workers to share emotions as well as providing compassion training not only can increase compassionate care toward patients and other staff but also may protect healthcare workers for empathic distress and burnout and impact on health system outcomes like costs [71–75]. There has been an emphasis globally on implementing compassionate care and its benefits in healthcare settings as well as how to improve uptake [76]. Further, healthcare worker burnout increased during the COVID-19 pandemic, but there have been few lasting changes to support healthcare worker wellbeing [77, 78]. Of particular importance in this study’s findings was the potential connection in the higher-retention facilities between healthcare worker cohesiveness and morale, patient perceptions of compassionate care, and high retention.

In accordance with Connect’s aesthetic improvement strategy, research has established the positive impact of physical environment and aesthetics on patient and staff wellbeing in healthcare settings [79–81]. This strategy was based on our finding that higher-retention facilities had more aesthetically pleasing and comfortable common areas, such as those with fresh paint, outdoor gardens, and motivational posters on the walls. However, no studies to our knowledge have examined the impact of physical environment on improved healthcare outcomes, such as retention on ART or VLS among PWH, or on staff wellbeing.

Within the domain of expediting workflow procedures, we found that the strategies we identified within higher-retention facilities (e.g., pre-pulling folders and implementing “Welcome Back Services”) were those already recommended in existing system-wide policies [3], but not implemented fully or at all in the lower-retention facilities. Learning that Connect parallels prior strategies or planned interventions validates our findings and emphasizes the need to incorporate implementation facilitation and to understand and document how Connect can be sustained, if it is found effective.

Several limitations of our study must be noted. Data collection from the six facilities to uncover “deviant” characteristics, or strategies of higher-retention facilities (i.e., those used by high-performing outliers)—coincided with the start of the COVID-19 pandemic, causing delayed access to the facilities, and may have affected perceptions of patient care. In addition, while our original goal was to only develop strategies based on findings from higher-retention facilities, we found that there were no single characteristics or strategies at these facilities exclusively. Nevertheless, findings incorporated in the intervention strategies, such as compassionate care, were found to a much greater extent at higher-retention facilities than in lower-retention facilities. An additional limitation is that we did not have sufficient data on the number and expertise of staff across all clinics. As noted earlier, low staff to patient ratios in public health clinics in South Africa are common. Low staffing could affect the patient and staff experience. In healthcare facilities in South Africa, data on staff skills and placement are often not accurately captured. For example, staff initially employed at one facility who move to another facility may only be recorded in their facility of initial employment. Further, staff sometimes oversee a range of facilities and thus are not recorded as working at a specific facility. Facilities also have access to staff from nongovernmental organizations, which is not recorded in the employment system. Thus, staffing information, while important, is not likely to be accurate. Further, staff feeling overworked was discussed at all facilities, despite differences in overall experiences and attitudes between higher- and lower-retention facilities. Given that low staffing is pervasive across all public health clinics, we designed our strategies to apply to clinics even where staffing and resources are low. Nevertheless, we plan to systematically collect real-time staffing data directly from clinics in a future, larger trial. Finally, our PD themes emerged from in-depth, composite analysis of qualitative data. In a future, larger trial, we will measure constructs quantitatively, including team cohesion, burnout, and compassion, and test effects of the Connect program on changes in these constructs and on ART retention. While our findings reflect rigorous qualitative work and suggest possible differences between higher- and lower-retention facilities, we acknowledge that there are a multitude of factors that influence ART retention in South Africa, and that no single intervention will resolve all challenges. Our intervention targeting improvement of staff and patient experiences, if effective in a larger trial, is one possible tool for improving care.

## Conclusions

A stakeholder-engaged study of characteristics and strategies of low-resource facilities in Cape Town, South Africa, that have above average retention rates for PWH on ART yielded a multicomponent intervention called Connect that emphasizes staff and patient wellbeing. This type of intervention complements data-driven approaches to improve retention, such as those set forth in Operation Phutuma [82], a national program in South Africa to improve care for PWH. Our stakeholders pointed out that study findings, with their focus on shifting from stress to compassion by recognizing common humanity, highlight elements of Ubuntu, a South African philosophy emphasizing common humanity and community [83], as does the Connect program itself. Our next steps are to evaluate preliminary effects of Connect on retention in HIV care, using data from a pilot study; if effective, Connect, with proper implementation support, may help low-resource facilities improve retention and outcomes for PWH within and outside of South Africa.

## Supplementary Information

Additional File 1: Table S1: Facility Characteristics (LR = lower retention, HR = higher retention)

Additional File 2: COREQ (COnsolidated criteria for REporting Qualitative research) Checklist

Additional File 3: Leadership Interview – Phase 1

Additional File 4: Provider Focus Group Guide – PHASE 1

Additional File 5: Table S2: Leadership Interview and Provider and Patient Focus Group Questions: Select Examples

Additional File 6: Patient Focus Group Guide – PHASE 1

Additional File 7: Patient Shadowing Form

Additional File 8: Codebook

## Data Availability

The qualitative data generated analyzed for the current study are not publicly available due to information that could compromise facility, staff or patient privacy.

## Abbreviations

ART: Antiretroviral therapy
ARV: Antiretroviral
CDC: Community day centers
CHC: Community health centers
COREQ: COnsolidated criteria for REporting Qualitative research
COVID-19: Coronavirus disease
HAST: HIV and AIDS, sexually transmitted infections, and tuberculosis
HIV: Human immunodeficiency virus
HR: Higher-retention facility
LR: Lower-retention facility
MSF: Department of Health South African and Médecins Sans Frontières
NIMH: National Institute of Mental Health
PD: Positive deviance
PhD: Doctor of philosophy
PWH: People with HIV
RNA: Ribonucleic acid
SAB: Stakeholder Advisory Board
UNAIDS: Joint United Nations Programme on HIV/AIDS
USD: United States dollar
VLS: Viral load suppression
WCDHW: Western Cape Department of Health and Wellness
