# Supplementary material for: Using positive deviance to enhance HIV care retention in South Africa: development of a compassion-focused program to improve the staff and patient experience
Source: BMC Glob Public Health. 2025 Feb 6;3:8. doi: 10.1186/s44263-025-00123-3 (PMC11800582; doi:10.1186/s44263-025-00123-3)
Supplement: Supplementary file 1 — Additional File 1: Table S1: Facility Characteristics (LR = lower retention, HR = higher retention) [file 44263_2025_123_MOESM1_ESM.pdf]

Table S1: Facility Characteristics (LR = lower retention, HR = higher retention)

Clinic	2018 12 - Month Retention Rate	2018 Size (# of patients)	Description, Setting
Facility 1 (HR)	73.9%	Small (156)	• 18,7 km from City Centre.<br>• An old, historic building surrounded by grass and trees.<br>• Situated in residential neighbourhood, on border between an upper middle class residential and commercial area.<br>• Draws patients from the local community and are also from across Cape Town.<br>• Close to major transport routes (busses, taxis, trains).<br>• Area is considered by staff to be safe, especially during business hours.
Facility 2 (LR)	52.0%	Small (208)	• 25,5 km from City Centre.<br>• A built-for-purpose facility.<br>• Situated in residential neighbourhood near shopping area, warehouses and state departments.<br>• Draws a mix of patients: nearby residents, others working in the area, including many people who are homeless and unemployed.<br>• Near large taxi rank, bus station, and train station.<br>• Area is viewed by staff as safe.
Facility 3 (HR)	71.9%	Medium (540)	• 25,5 km from City Centre.<br>• A built-for-purpose facility.<br>• Situated in a poor, working-class area.<br>• Draws patients from poor areas and informal settlements.<br>• Not close to transport routes, but accessible from surrounding communities; close to taxi and bus route.<br>• High gang membership and crime levels.
Facility 4 (LR)	56.3%	Medium (308)	• 24,2 km from City Centre.<br>• Built-for-purpose facility.<br>• Situated in poor working-class area.<br>• Situated in a warehouse and wholesale business area, with residential space around.<br>• Near a train station and taxi routes.<br>• Area is viewed by staff as safe during the day (gangs are a risk at night).
Facility 5 (HR)	71.8%	Large (993)	• 29,8 km from the City Centre.<br>• A built-for-purpose facility.<br>• Situated in poor working-class township.

			• Draws a large mix of patients with a high number of poor.<br>• Near large taxi rank and bus station.<br>• Staff report that violence and theft are common in the area.
Facility 6 (LR)	59.2%	Large (992)	• 23,6 km from City Centre.<br>• A built-for purpose facility.<br>• Located near a shopping centre, surrounded by commercial property and high-density residential property.<br>• The area is close to an informal settlement.<br>• Draws patients mainly from informal settlements.<br>• Area considered by staff to be unsafe, with reports of robberies.
