# Supplementary material for: Using positive deviance to enhance HIV care retention in South Africa: development of a compassion-focused program to improve the staff and patient experience
Source: BMC Glob Public Health. 2025 Feb 6;3:8. doi: 10.1186/s44263-025-00123-3 (PMC11800582; doi:10.1186/s44263-025-00123-3)
Supplement: Supplementary file 3 — Additional File 3: Leadership Interview – Phase 1 [file 44263_2025_123_MOESM3_ESM.pdf]

# Leadership Interview Guide – Phase 1

| | |
|---|---|
| **Date:** | ____/____/____ |
| **Clinic:** | |
| **Clinic Type:** | ☐ Higher-retention<br>☐ Lower-retention |
| **Leader Type:** | ☐ Medical Leadership<br>☐ Administrator |
| **Investigator:** | |
| **Note-Taker:** | |
| **Start Time:** | |
| **End Time:** | |
| **# Attendees:** | |

## INTRODUCTIONS AND CONSENT

Hi. My name is [**NAME**] and this is [**NAME**]. We are from the Human Sciences Research Council (HSRC). HSRC is an organization appointed by government to conduct research to improve health and well-being. We are conducting a research study to learn more about how to improve care for people living with HIV.

Today we are interested in hearing your thoughts about services for people living with HIV at [**CLINIC**]. Before we begin I'd like to give you the study consent form. The form explains the study. If you agree to participate in this interview, please sign at the bottom.

We realize this past year has been challenging due to the COVID pandemic. We'll be asking some questions about how things are here generally, and what kinds of changes there have been since the start of the pandemic.

**INVESTIGATOR: GIVE THE CONSENT FORM. ASK THE PARTICIPANT TO READ THROUGH THE FORM. SUMMARIZE THE FORM OUT LOUD. ASK THE PARTICIPANT TO SIGN IF THEY ARE WILLING TO PARTICIPATE AND BE RECORDED. ASK IF THEY HAVE ANY QUESTIONS. COLLECT THE FORM.**

Your role is to answer the questions as best you can. There are no right or wrong answers. You do not have to answer any question that you do not want to answer. I will be leading today's interview. [**NAME**] will be taking notes.

We would like to record this session to make sure we don't miss anything important. We will use the information from the group to make presentations and write reports and articles about the project. In the presentations, reports, and articles, we will never use your name or anything that might identify you. Do you have any questions?

Before we begin, we'll ask you to complete a brief survey. Please do not put your name on the survey.

# Brief Survey

**(Please do not put your name on this survey)**

**What is your current role at the clinic? (both professional role and service area of clinic)**

__________________________

**How long have you been working at the clinic?**

_______ years _______ months

**For how long have you been working in your current role at this clinic?**

_______ years _______ months

**What gender do you identify with?**

______ Female

______ Male

______ Trans/Nonbinary

**What is your current age? ______**

**In your opinion, what do you think are the top two reasons people living with HIV stay in care at this clinic?**

**What are the top two reasons people living with HIV do not stay in care at this clinic?**

# INTERVIEW QUESTIONS

**TURN ON RECORDER**

1. **First, I'd like to get a bit of background about the clinic. Please tell me about the services offered here, the patients, and the providers**

   PROBE:

   - Is the clinic a stand-alone ARV clinic or is it integrated? What other services are provided? (*Integrated = provides a wide range of services. Be aware that not all clinics think of themselves as integrated even though they might be.*)
     PROBE:
     - *How do these services support each other?*
     - *How well are community health workers integrated*
   - How long has the clinic been providing ARVs to people living with HIV?
   - What outside (*outside of WCDOH*) supports and resources has the clinic received in the past?
     PROBE:
     - *Research studies outside of clinics?*
     - *NGO/CBO support (e.g., treatment action campaign); those set up around COVID*
     - *University support systems (e.g., UCT social workers)*

2. **I'd like to understand how clinic protocols and procedures for people living with HIV, from when they are tested, referred for treatment, and begin on ARVs. So, from when they first arrive at the clinic, tell me about their process. (And here, I want to know about how things are now, but also how they were before COVID.)**

   PROBE:
   - Who is the first contact at the clinic? What are those interactions?
     PROBE:
     - *Do they interact with a security guard first? What is the role of the security guard? What happens when patients interact with the security guard? Would you describe this interaction between the patient and security guard as generally friendly or unfriendly?*

- *What about the receptionist? What is their role? What happens when patients interact with the receptionist? Would you describe this interaction between the patient and receptionist as generally friendly or unfriendly?*
  - *What about other staff? Community health worker? Others?*
- What is the waiting room context?
  PROBE:
  - *Water fountain?*
  - *TV?*
  - *Coffee?*
  - *Other*
- Tell me about testing (Who, where, when?)
- Are most patients who get treatment at this clinic also tested here?
  - [IF NO] Where are they usually tested? How are they referred here?
- What happens after a positive test? (Who discusses results, how is the next visit scheduled? Confirmatory tests?)
- What is the typical amount of time between testing and ARV initiation? (*Be sure to ask about antenatal v. not antenatal patients; TB v. non-TB*)
- What is the difference in procedures if someone arrives saying that they have tested HIV+ and need to start treatment compared with if someone tests positive at the clinic for the first time?
- Are there any components particular to this process that we may have missed?

3. **I want to acknowledge that COVID may have had an impact on how things work here. (*If not already discussed above.*)**

- How did the COVID-19 pandemic change clinic protocols and procedures for people living with HIV?
- How much have things changed in the clinic from how things were before the pandemic?
- What has improved here since COVID?
- Are there lessons from COVID that may help improve longer-term adherence?

4. **Next, I'd like to know more about how clinic visits go for people living with HIV after they start on ARVs. Please describe the workflow for people living with HIV at [CLINIC]. What I mean by workflow is what happens for people with HIV from when they get to the clinic for their appointment until**

**they leave? Please explain visits in which they are just picking up medication and also visits in which they see the doctor.**

PROBE:
- Who checks in patients with HIV? Are they greeted by the same person every time?
- How long do people living with HIV usually wait during their visit to see a doctor or nurse (if they have a full appointment scheduled)? Is this the same as for other patients who are not living with HIV (*if applicable*)? How have you address this in your clinic?
- In what way do procedures change when the clinic gets even busier than usual?
- How convenient or not convenient are the clinic hours for patients? How well do the hours work for providers specifically? For patients? (Work well? Don't work well?)
- In what ways is the clinic workflow different now from how it was before the COVID-19 pandemic?

5. **Retaining patients in care after they begin ARVs is challenging for most clinics. What are the top three reasons people with HIV do not stay in care at this clinic?**

PROBE (after they provide the top 3):
- Patients' work schedules/transfers?
- Clinic hours?
- Transportation?
- Family/childcare?
- Problems with staff?
- Certain aspects of the clinic?
    - Low funding?
    - Low morale?
    - Understaffing?
    - Space issues?
    - Privacy issues?
- Lifestyle issues (drugs, alcohol, mental health issues, community violence, COVID)
- Stigma from other patients at the clinic?
- Stigma from providers at the clinic?
- In what ways did the pandemic change retention in care for people with HIV?

6. **Even though retaining patients in care is difficult, there are patients who stay in care. Tell me what you think has worked well for helping patients living with HIV stay in care at this clinic? [FOR ALL QUESTIONS, PROBE FOR MORE DETAILS. "TELL ME MORE." "WHAT IS HELPFUL ABOUT X PROGRAM? HOW DOES X PROGRAM WORK?]**

   PROBE:

- Stability of staff, with regard to turnover?
- Qualities of staff at this clinic?
  - Good/high morale?
    - [IF YES]: How do staff manage to have high morale at this clinic?
  - A specific staff member or staff members?
    - [IF YES]: What does this staff member(s) do in particular that might help patients with HIV stay in care?
- Dedicated providers?
  - Patients staying with the same provider for all of their care?
  - Staff member dedicated to patient monitoring and follow-up?
  - Retention/adherence champions? (Champions are people who have a lot of training in retention and adherence and are specifically focused on keeping patients in care and on ARVs.)
  - Community health workers/peers?
  - Community lay counsellors
- Special trainings for providers?
- Special programs for people on ARVs? FOR EACH: When are patients eligible for them? What are the details of the organizations and range of impacts? How do they work for people on ARVs? (
  - Adherence clubs
  - Treatment buddies
  - Vans for transportation?
  - Special ways to pick up medications, such as at community pick-up points?
- Special policies or protocols for people on ARVs?
  - Flexible appointment times?
- Special funding given to the clinic to help retain people on ARVs?
  - Retention as a funding priority?
  - Special funding streams?
  - DoH wide campaigns and programmes? How do these integrate?
- Benchmarks or metrics for keeping people in care?
  - Clinic benchmarks? Retention targets?

    - Local benchmarks?
    - National benchmarks?
- Using data to help people in care?
    - Data to track people lost to follow-up?
    - Data to provide clinic with feedback on retention rates?
- Clinic space?
    - A dedicated waiting room?
    - Other things that increase privacy?
    - Special amenities to make people feel welcome?
- Relationships with other agencies and professionals?
    - Communication with mental health and substance use specialists?
    - Access to specialists for consultation?

- [IF THERE ARE SPECIAL PROGRAMS.] Are they usually sustained? How does the clinic manage to sustain special programs?

- [IF THERE IS PRIVATE CLINIC AREA, LIKE A SEPARATE WAITING AREA OR ENTRY FOR PATIENTS LIVING WITH HIV]
    - How well does the private area waiting area or entry work? How well does it work for providers specifically? For patients? (Works well? Doesn't work well?)
    - Are there any signs indicating this separate area? Impact of signs if any?

- Other things about the clinic, providers, or patients that could be making a difference, even if it is a small thing like giving food on certain days?

- What about ways people were retained in the clinic during the pandemic?

- Is there anything else that we haven't discussed that you think could be helping this clinic keep a patients with HIV care? Think of anything, even if something very small, that you think might be helping.

7. **Next, I have some questions about the work culture at this clinic. [FOR ALL POSITIVE RESPONSES, ASK QUESTIONS ABOUT HOW THE CLINIC MANAGES TO SUSTAIN THAT.]**

   PROBE:

   - How would you describe the morale at this clinic, generally?

- How about relationships between staff members?
- Teamwork?
- How would you describe your relationship to the WCDoH management structure?
- In what ways did the culture here change as a result of the pandemic?

**8. To what extent is HIV stigma an issue at this clinic?**

PROBE:

– In what ways is it an issue?
– Does the clinic address HIV stigma?
– What kinds of things may still make patients with HIV feel stigmatized?
– Is there anything special at this clinic that may be helping patients not to feel stigmatized?
– How is this/can this be addressed by the clinic? Support provided?

**9. We're just about finished with the discussion. Do you have any last thoughts about what could be helping people with HIV stay in care at this clinic in particular? Is there anything that we may have missed?**

Thank you for your time. I'm going to turn off the recorder. [**TURN OFF RECORDER.**]
