# Supplementary material for: Using positive deviance to enhance HIV care retention in South Africa: development of a compassion-focused program to improve the staff and patient experience
Source: BMC Glob Public Health. 2025 Feb 6;3:8. doi: 10.1186/s44263-025-00123-3 (PMC11800582; doi:10.1186/s44263-025-00123-3)
Supplement: Supplementary file 4 — Additional File 4: Provider Focus Group Guide – PHASE 1 [file 44263_2025_123_MOESM4_ESM.pdf]

PROVIDER FOCUS GROUP GUIDE – PHASE 1

**Date:**	____/____/____
**Clinic:**	
**Clinic Type:**	☐ Higher-retention<br>☐ Lower-retention
**Provider Type:**	☐ Medical staff group<br>☐ Lay staff group
**Investigator:**	
**Note-Taker:**	
**Start Time:**	
**End Time:**	
**# Attendees:**	

INTRODUCTIONS AND CONSENT

Hi. My name is [**NAME**] and this is [**NAME**]. We are from the Human Sciences Research Council (HSRC). HSRC is an organization appointed by government to conduct research to improve health and well-being. We are conducting a research study to learn more about how to improve care for people living with HIV.

Today we are interested in hearing your thoughts about services for people living with HIV at [**CLINIC**]. Before we begin I'd like to give out the study consent form. The form explains the study. If you agree to participate in this group, please sign at the bottom. If you don't want to participate you are not required to stay.

We realize this past year has been challenging due to the COVID pandemic. We'll be asking some questions about how things are here generally, and what kinds of changes there have been since the start of the pandemic.

INVESTIGATOR: HAND OUT THE CONSENT FORM. ASK PARTICIPANTS TO READ THROUGH THE FORM. SUMMARIZE THE FORM OUT LOUD. ASK PARTICIPANTS TO AND SIGN IF THEY ARE WILLING TO PARTICIPATE AND BE RECORDED. ASK IF THEY HAVE ANY QUESTIONS. COLLECT THE FORM.

Your role in this group is to answer the questions as best you can. There are no right or wrong answers. You do not have to answer any question that you do not want to answer. I will be leading today's focus group. [**NAME**] will be taking notes and assisting me when needed.

We would like to record this session to make sure we don't miss anything important. We will use the information from the group to make presentations and write reports and articles about the project. In the presentations, reports, and articles, we will never use your name or anything that might identify you.

To further protect your privacy, we have given you each a card with a number. We will refer to you by your number instead of your name during the group. Please put the card on the table in front of you.

Do you have any questions?

Before we begin, we'll ask you to complete a brief survey. Please do not put your name on the survey.

Brief Survey

(Please do not put your name on this survey)

What is your current role at the clinic? (both professional role and service area of clinic)

______ Administrator

______ Physician

______ Nurse

______ Lay Counsellor

______ ART Clerk

______Other staff (Specify role): ______________________________

How long have you been working at the clinic?

_______ years _______ months

For how long have you been working in your current role at this clinic?

_______ years _______ months

What gender do you identify with?

______ Female

______ Male

______ Trans/Nonbinary

What is your current age? ______

In your opinion, what do you think are the top two reasons people living with HIV stay in care at this clinic?

What are the top two reasons people living with HIV do not stay in care at this clinic?

GROUND RULES

Before we start the discussion, I'd like to go over a few ground rules.

- My role as moderator will be to guide the discussion.
- [**NAME**] will be taking notes.
- Cell phones
  - Please turn off your phones
  - If you cannot and if you must respond to a call, please leave the room and do so as quietly as possible and rejoin us as quickly as you can
- One person speaks at a time – it makes it easier to hear what is on the recording.
- There are no right or wrong answers, only differing points of view
- You don't need to agree with others, but please listen respectfully as others share their views
- Feel free to talk to each other, not just to me
- What is said in the room stays in the room
- Please do not mention each other's names or mention others by name who are not here

FOCUS GROUP QUESTIONS

TURN ON RECORDER

1. **First, I'd like to know more about how clinic visits go for people living with HIV after they start on ARVs. Please describe the workflow for people living with HIV at [CLINIC]. What I mean by workflow is what happens for people with HIV from when they get to the clinic for their appointment until they leave, including getting tests and picking up medication.**

   PROBE (ASK ALL NOT BROUGHT UP BY PARTICIPANTS):
   - How long do people living with HIV usually wait during their visit to see a doctor or nurse? Is this the same as for other patients who are not living with HIV?
   - How convenient or not convenient are the clinic hours for patients? How well do they work for providers specifically? For patients? (Works well? Doesn't work well?)
   - Who checks in patients with HIV? Are they greeted by the same person every time?
   - In what way do procedures change when the clinic gets even busier than usual?
   - Waiting time is often a concern for patients. How have you addressed this in your clinic?
   - Are there any components particular to this process that we may have missed?

2. **I want to acknowledge that COVID may have had an impact on how things work here.**

   - How did the COVID-19 pandemic change clinic protocols and procedures for people living with HIV?
   - How much have things changed in the clinic from how things were before the pandemic?
   - In terms of how things are now, is it the same or different from how it was before the pandemic?

3. **Retaining patients in care after they begin ARVs is challenging for most clinics. What are the most common reasons people with HIV do not stay in care at this clinic?**

   PROBE (ASK ALL NOT BROUGHT UP BY PARTSICIPANTS):
   - Work schedules/transfers?
   - Clinic hours?
   - Transportation?
   - Family/childcare?
   - Problems with staff?
   - Certain aspects of the clinic?
     - Low funding?
     - Low morale?
     - Understaffing?
     - Space issues?
     - Privacy issues?
   - Lifestyle issues (drugs, alcohol, mental health issues, community violence, COVID)
   - Stigma from other patients at the clinic?
   - Stigma from providers at the clinic?

   - What would you say are the top three reasons patients don't stay in care at this clinic?

   - In what ways did the pandemic change retention in care for people with HIV?

4. **Even though retaining patients in care is difficult, there are patients who stay in care. Tell me what you think works well for helping patients living with HIV stay in care at this clinic? [FOR ALL QUESTIONS, PROBE FOR MORE DETIALS. "TELL ME MORE." "WHAT IS HELPFUL ABOUT X PROGRAM? HOW DOES X PROGRAM WORK?]**

   PROBE (ASK ALL NOT BROUGHT UP BY PARTICIPANTS):
   - Qualities of staff at this clinic?
     - Good/high morale?
       - [IF YES]: How do staff manage to have high morale at this clinic?
     - A specific staff member or staff members?

        - [IF YES]: What does this staff member(s) do in particular that might help patients with HIV stay in care?

- Dedicated providers?
    - Patients staying with the same provider for all of their care?
    - Staff member dedicated to patient monitoring and follow-up?
    - Retention/adherence champions? (Champions are people who have a lot of training in retention and adherence and are specifically focused on keeping patients in care and on ARVs.)
    - Community health workers/peers?
    - Community lay counsellors?
- Special trainings for providers?
- Special programs for people on ARVs? FOR EACH: When are patients eligible for them? What are the details of the organizations and range of impacts? How do they work for people on ARVs?
    - Adherence clubs
    - Treatment buddies
    - Vans for transportation?
    - Special ways to pick up medications such as at community pick-up points?
- Special policies or protocols for people on ARVs?
    - Flexible appointment times?
- Special funding given to the clinic to help retain people on ARVs?
    - Retention as a funding priority?
    - Special funding streams?
    - DoH wide campaigns and programmes? How do these integrate?

- Benchmarks or metrics for keeping people in care?
    - Clinic benchmarks? Retention targets?
    - Local benchmarks?
    - National benchmarks?
- Using data to help people in care?
    - Data to track people lost to follow-up?
    - Data to provide clinic with feedback on retention rates?
- Clinic space?
    - A dedicated waiting room?
    - Other things that increase privacy?
    - Special amenities to make people feel welcome?
- Relationships with other agencies and professionals?
    - Communication with mental health and substance use specialists?
    - Access to specialists for consultation?

- [IF THERE ARE SPECIAL PROGRAMS.] How does the clinic manage to sustain special programs?

- [IF THERE IS PRIVATE CLINIC AREA, LIKE A SEPARATE WAITING AREA OR ENTRY FOR PATIENTS LIVING WITH HIV]
    - o How well does the private area work How well does it work for providers specifically? For patients? (Works well? Doesn't work well?
    - o Are there any signs indicating this separate area? Impact of signs if any?

- Other things about the clinic, providers, or patients that could be making a difference?

- In what ways did the pandemic change retention in care for people with HIV?

- Is there anything else that we haven't discussed that you think could be helping this clinic keep patients with HIV in care? Think of anything, even if something very small, that you think might be helping.

5. Tell me about what it's like to work here – about the work culture. [FOR ALL POSITIVE RESPONSES, ASK QUESTIONS ABOUT HOW THE CLINIC MANAGES TO SUSTAIN THAT.]

PROBE (ASK ALL NOT BROUGHT UP BY PARTICIPANTS):

- How would you describe morale generally?
- How about relationships between staff members?
- Teamwork?
- In what ways did the culture here change as a result of the pandemic?

6. To what extent is HIV stigma an issue at this clinic?

PROBE:
– In what ways is it an issue?
– Does the clinic address HIV stigma?

- What kinds of things may still make patients with HIV feel stigmatized?
- How is this/can this be addressed by the clinic? Support provided?

7. **We’re just about finished with the discussion. Do you have any last thoughts about what could be helping people with HIV stay in care at this clinic in particular? Is there anything that we may have missed?**

Thank you for your time. I’m going to turn off the recorder. [**TURN OFF RECORDER.**]
