# Supplementary material for: Using positive deviance to enhance HIV care retention in South Africa: development of a compassion-focused program to improve the staff and patient experience
Source: BMC Glob Public Health. 2025 Feb 6;3:8. doi: 10.1186/s44263-025-00123-3 (PMC11800582; doi:10.1186/s44263-025-00123-3)
Supplement: Supplementary file 5 — Additional File 5: Table S2: Leadership Interview and Provider and Patient Focus Group Questions: Select Examples [file 44263_2025_123_MOESM5_ESM.pdf]

Table S2: Leadership Interview and Provider and Patient Focus Group Questions: Select Examples

Domain	Examples
**Retention**	*Even though retaining patients in care is difficult, there are patients who stay in care. Tell me what you think works well for helping patients living with HIV stay in care at this clinic.*
**Workflow**	*Please describe the workflow for people living with HIV at [FACILITY NAME]. What I mean by workflow is what happens for people with HIV from when they get to the clinic for their appointment until they leave, including getting tests and picking up medication.*
**Workplace Climate**	*Tell me about what it's like to work here – about the work culture.*
**PD Probes**	*Many facilities have difficulties retaining PWH in care. How has this clinic overcome these challenges? What one or two specific aspects of this clinic help patients to keep coming back here?*
**Capacity-specific probes**	*What policies or procedures, if any, could be helping with retention of PWH? What practices or data systems does the clinic use to help track and contact PWH who fall out of care? How are staff supported in doing their jobs here?*
**Patient questions and PD probes**	*Tell me about your experience receiving care for your HIV at this clinic. What makes you feel welcome (or unwelcome) here? A lot of patients don't come back after they start treatment for their HIV; what has helped you to stay in care here?*
