# Supplementary material for: Using positive deviance to enhance HIV care retention in South Africa: development of a compassion-focused program to improve the staff and patient experience
Source: BMC Glob Public Health. 2025 Feb 6;3:8. doi: 10.1186/s44263-025-00123-3 (PMC11800582; doi:10.1186/s44263-025-00123-3)
Supplement: Supplementary file 6 — Additional File 6: Patient Focus Group Guide – PHASE 1 [file 44263_2025_123_MOESM6_ESM.pdf]

PATIENT FOCUS GROUP GUIDE – PHASE 1

**Date:**	____/____/____
**Clinic:**	
**Clinic Type:**	☐ Higher-retention<br>☐ Lower-retention
**Focus Group Gender:**	☐ Female<br>☐ Male
**Investigator:**	
**Note-Taker:**	
**Start Time:**	
**End Time:**	
**# Attendees:**	

INTRODUCTIONS AND CONSENT

Hi. My name is [**NAME**] and this is [**NAME**]. We are from the Human Sciences Research Council (HSRC). HSRC is an organization appointed by government to conduct research to improve health and well-being. We are conducting a research study to learn more about how to improve care for people living with HIV.

Today we are interested in hearing your thoughts about services for people living with HIV at [**CLINIC**]. Before we begin, I'd like to give out the study consent form. The form explains the study. If you agree to participate in this group, please sign at the bottom. If you don't want to participate you are not required to stay.

INVESTIGATOR: HAND OUT THE CONSENT FORM. ASK PARTICIPANTS TO READ THROUGH THE FORM. READ THE FORM OUT LOUD. ASK PARTICIPANTS TO AND SIGN IF THEY ARE WILLING TO PARTICIPATE AND BE RECORDED. ASK IF THEY HAVE ANY QUESTIONS. COLLECT THE FORM.

Your role in this group is to answer the questions as best you can. There are no right or wrong answers. You do not have to answer any question that you do not want to answer. I will be leading today's focus group. [**NAME**] will be taking notes and assisting me where needed.

We would like to record this session to make sure we don't miss anything important. We will use the information from the group to make presentations and write reports and articles about the project. In the presentations, reports, and articles, we will never use your name or anything that might identify you.

To further protect your privacy, we have given you each a card with a number. We will refer to you by your number instead of your name during the group. Please put the card on the table in front of you.

Do you have any questions?

Before we begin, we'll ask you to complete a brief survey. Please do not put your name on the survey.

Brief Survey

(Please do not put your name on this survey)

How old are you? ______

What gender do you identify with?

______ Female

______ Male

______ Trans/Nonbinary

What is your race?

______ Black

______ Colored/Mixed Race

______ White

______ Asian

______ Other race, specify: ____

What month and year were you told you had HIV?

_______ Month _______ Year

What month and year were did you start on ART?

_______ Month _______ Year

ONLY ANSWER THE NEXT FEW QUESTIONS IF YOU ARE A WOMAN

Are you currently pregnant?

No _____ Yes _____

Did you recently give birth?

No _____ Yes _____

If you recently gave birth, on what date was your baby born?

_______ Month _______Day _______ Year

About how long have you been receiving care at this clinic?

_______ Days or _______ Months or _______Years

GROUND RULES

Before we start the discussion, I'd like to go over a few ground rules.

- My role as moderator will be to guide the discussion.
- [**NAME**] will be taking notes.
- Cell phones
  - Please turn off your phones
  - If you cannot and if you must respond to a call, please leave the room and do so as quietly as possible and rejoin us as quickly as you can
- One person speaks at a time – it makes it easier to hear what is on the recording.
- There are no right or wrong answers, only differing points of view
- You don't need to agree with others, but please listen respectfully as others share their views
- Feel free to talk to each other, not just to me
- What is said in the room stays in the room.
- Please do not mention each other's names or mention others by name who are not here. This is very important for helping us protect everyone's privacy.

FOCUS GROUP QUESTIONS

TURN ON RECORDER

1. **To start, tell me what it's like for people living with HIV to visit this clinic.**

   PROBE (ASK ALL):
   - How welcoming do you feel this clinic is for people living with HIV?
   - What might make people living with HIV feel welcome at this clinic?
   - What might make people living with HIV feel less welcome at this clinic?
   - How much do you feel that patients get good care here? Why or why not?
   - Why did you think patients with HIV choose to go to this clinic, instead of another clinic?

In what ways did getting care for HIV at this clinic change during the COVID-19 pandemic?

2. **Next, I'd like to know more about how visits go for people with HIV at this clinic. Can you walk me through the steps of what happens from when you get here, to seeing the provider, getting any medications, and getting any tests done, until when you leave?**

   PROBE (ASK ALL NOT BROUGHT UP BY PARTICIPANTS):
   - Who do you talk to first? What's that like? Is it the same person every time? Do you feel like the staff know you? How friendly or unfriendly are they?
   - How long do you usually wait for your appointment?
   - How convenient or not convenient are the HIV clinic hours? Why or why not?
   - [IF THERE A SEPARATE CLINIC AREA, WAITING AREA, OR ENTRY FOR PATIENTS LIVING WITH HIV]
     - What do you think about the clinic having a separate area for people living with HIV? How much does it work well, or does not work well? Why or why not? How does it make patients with HIV feel?
   - Do you see the same provider each visit, or do you see different providers? How do you feel about that? How much would you prefer to see the same [or different] providers? Why or why not?
   - Where do you pick up your medication? For example, primarily at the clinic, or from clubs or other pick-up sites? How does that go for you?
     - [IF CLINIC-BASED]

        - How do you feel about picking up your medication at the clinic? How helpful would it be to pick your medications up at a community pick-up point, like a specific location in your community?
    - [IF COMMUNITY PICK-UP POINTS]
        - How do you feel about community pick-up points for medications? How often do you use them?

- Which parts of the clinic visit are hardest for you, for example, checking in, seeing the provider, getting tests done, or picking up medications? Why?

- Which parts of the visit are easiest? What's good about them?

3. Staying on ARVs is hard for many patients. What has helped you stay in care? What has helped at this clinic?

PROBE (ASK ALL NOT BROUGHT UP BY PARTICIPANTS):

- Clinic environment?
    - [IF YES]: What about the clinic environment? Friendly? Clean? Short wait times?
- Qualities of staff at this clinic?
    - Friendly?
    - Helpful?
    - Communicate well?
- Relationship with one or more of the staff here?
    - [IF YES]: What does this staff member(s) do in particular that helps you come back to the clinic?
- Special programs for people on ART, like adherence clubs?
    - [IF YES]: Tell me more. What are these programs? What about them is helpful? When did you start using them? How often do you go? What services do they offer? Do you go to one specific club? For what services do you still go to the clinic?
- Community health workers?
    - [IF YES]: Tell me more. What about having community workers is helpful?
- Provision of resources and tools to assist pregnancy \ childcare?
    - [IF YES]: Tell me more. What are these resources and tools and how have they helped?

4. **What kinds of things make it hard to come to your visits at this clinic?**

   PROBE (ASK ALL NOT BROUGHT UP BY PARTICIPANTS):
   - Work schedule?
   - Clinic hours?
   - Transportation?
   - Family/childcare?
   - Certain qualities of the clinic?
     - Unfriendly staff?
     - Crowded/long wait times?
   - Lifestyle issues (drugs, alcohol)
   - Depression or other emotional issues?
   - Stigma from other patients at the clinic?
   - Stigma from providers at the clinic?

How about during the pandemic?

5. **Even with these problems you mentioned, like [LIST PROBLEMS], you've still managed to stay in care here. Why do you think that you have been able to stay in care? What has the clinic done, if anything, that has helped you to stay in care? What could the clinic do better to help you to stay in care? Did this clinic do anything special during the pandemic that helped you to stay in care?**

6. **People living with HIV sometimes feel like they are stigmatized at clinics and by healthcare providers—are there things that make you feel bad when you get treatment here? Can you describe these? Do you feel like you are being judged or discriminated against for other reasons?**

7. **What does this clinic do, if anything, to help address HIV stigma?**

8. **We're just about finished with the discussion. Do you have any last thoughts about what could help other people with HIV stay in care at this clinic?**

Thank you for your time. I'm going to turn off the recorder. [**TURN OFF RECORDER.**]
