# Supplementary material for: Using positive deviance to enhance HIV care retention in South Africa: development of a compassion-focused program to improve the staff and patient experience
Source: BMC Glob Public Health. 2025 Feb 6;3:8. doi: 10.1186/s44263-025-00123-3 (PMC11800582; doi:10.1186/s44263-025-00123-3)
Supplement: Supplementary file 7 — Additional File 7: Patient Shadowing Form [file 44263_2025_123_MOESM7_ESM.pdf]

**DATE**	____/____/____
**CLINIC**	
**INVESTIGATOR**	

**PATIENT GENDER**	
**PATIENT AGE**	
**LENGTH OF TIME IN CARE**	

PD Shadowing Form

TIME	LOCATION	SPACE, CLIMATE DESCRIPTION	PROVIDER TOUCH POINT; CARE EXPERIENCE	SHADOWER IMPRESSIONS	PATIENT IMPRESSIONS (POST-VISIT)
SAMPLE 7:00 AM	*Outside of Clinic, Front door*	*Outdoor area clean*	*Patient waiting outside for clinic to open with other patients; receptionist unlocks door on time*	*Receptionist friendly; greets all patients by name; warm*	*Pt glad doors opened on time, had been worried about work*

TIME	LOCATION	SPACE,<br>CLIMATE DESCRIPTION	PROVIDER TOUCH POINT;<br>CARE EXPERIENCE	SHADOWER IMPRESSIONS	PATIENT IMPRESSIONS<br>(POST-VISIT)

***After the visit, ask the patient:**

1. **How did this visit go for you?**
   a. **Did you feel welcome at this clinic? What made you feel more/less welcome?**
   b. **Were you comfortable with staff? With clinicians?**
2. **What went well during this visit? What didn't go as well?**
3. **Is this how visits at this clinic typically go for you?**
   a. **IF NO: How do they typically go?**
4. **Describe if there is anything you wish the staff or clinicians would have done differently today.**
5. **Tell me if there was anything that happened today or during past visits that keeps you coming back to this clinic for your ARVs?**
6. **In what ways is this clinic different from other clinics you've been to?**
