# Supplementary material for: Using positive deviance to enhance HIV care retention in South Africa: development of a compassion-focused program to improve the staff and patient experience
Source: BMC Glob Public Health. 2025 Feb 6;3:8. doi: 10.1186/s44263-025-00123-3 (PMC11800582; doi:10.1186/s44263-025-00123-3)
Supplement: Supplementary file 8 — Additional File 8: Codebook [file 44263_2025_123_MOESM8_ESM.pdf]

	Parent Code		Child Code	Definition
1	**Clinic Governance, Physical Structure, Resources, Environment**	a	Clinic services offered; governance; policies	Services offered. Clinic leadership. Policies regarding retention.
		b	Patient pathway through clinic	Patient pathway through the clinic.
		c	Equipment, furniture, toilets (infrastructure)	Comments or observations about equipment and furniture.
		d	Amenities for patients	Services and comforts are available to patients.
		e	Surrounding community	Area around the clinic.
		f	Stigma - Community	Presence of community stigma.
		g	Access to clinic	Clinic access, structural setup.
2	**Clinic Workforce**	a	Morale, burnout, cohesion	How clinic staff is doing/feeling. Statements about staff morale, burnout, cohesion.
		b	Management	Staff feelings about management. Managers are doing a good/bad job.
		c	Improvement programs	Programs benefits specifically to improve staff well-being or to improve staff-patient interactions.
		d	Staff selection and roles	Staff roles, critieria for selection, sustainability of staff.
		e	Clinic champion	Presence of core person or persons that stands out, who drives the ART program.
		f	NGOs	Presence and contributions of NGOs.
3	**Staff-to-Patient Relationships**	a	Staff respectfulness	Statements about kindness, respectfulness of staff toward patients.
		b	Patient-centered personalized care; compassion	Statements about staff passion for their job, for patients, going the extra mile.
		c	Stigma - Staff	Stigmatizing behaviors and/or comments by staff toward patients.

4	**Clinic Procedures**	**a**	Wait times	Any comments about wait times.
		**b**	Administrative procedures & workflow	Administrative/workflow procedures clinics follow/patients go through to obtain services, including bloods, pickups, etc.).
		**c**	Managing LTFU; non-adherence	Procedures for patients who are lost to follow up.
		**d**	Transfer/referral	Procedures related to patient transfers and referrals.
		**e**	Stigma - Procedural	Procedureal issues at clinic which increase or decrease stigma.
5	**Patient Characteristics & Behaviors**	**a**	Sociodemographics	Comments about patient socio-economic status, poverty, gender, race.
		**b**	ARV treatment experience and history	Patient history and and experience of HIV care.
		**c**	Behavioral health	Comments about patient substance use and mental health issues.
		**d**	Behavior toward staff	How staff treat patients.
		**e**	Mobility	Comments about patient mobility and reasons for transfer.
6	**Patient Support Programs (Formal or informal)**	**a**	Differentiated service delivery	Different programs and services for people at different phases of treatment.
		**b**	WhatsApp groups	Any comments about WhatsAPP groups specifically.
		**c**	Welcome back services	Any comments about Welcome Back Services.
		**d**	Behavioral health/social work services for patients	Mental health/social service, substance use services available to patients.
7	**COVID**			Comments about the impact of COVID.
8	**Potential Retention Facilitator or Barrier**			
		**a**	Potential Retention Facilitator	Comments about what keeps patients coming back to clinic for ART.
		**b**	Potential Retention Barrier	Comments about what keeps patients from coming back to clinic for ART.
9	**Recommendations for Improving Retention**			Include all recommendations for improving retention.
